# β-Carboline-α-aminophosphonate Derivative: A Promising Antitumor Agent for Breast Cancer Treatment

**DOI:** 10.3390/molecules28093949

**Published:** 2023-05-08

**Authors:** Caroline Pinto Zani, Aline Pinto Zani, Cristiane Melissa Thomazini, Karina Miyuki Retamiro, Aline Rufino de Oliveira, Débora Laís Gonçalves, Maria Helena Sarragiotto, Francielle Pelegrin Garcia, Sueli de Oliveira Silva, Celso Vataru Nakamura, Tania Ueda-Nakamura

**Affiliations:** 1 Laboratory of Technological Innovation in the Development of Pharmaceuticals and Cosmetics, State University of Maringá, Maringá CEP 87020-900, Paraná, Brazil; pg403171@uem.br (C.P.Z.); tunakamura@uem.br (T.U.-N.); 2 Department of Chemistry, State University of Maringá, Maringá CEP 87020-900, Paraná, Brazil

**Keywords:** breast cancer, MCF-7, MDA-MB-231, apoptosis, autophagy

## Abstract

Breast cancer is the most common type of cancer and the leading cause of cancer mortality among women worldwide. Considering the limitations of the current treatments available, we analyzed the in vitro cytotoxic potential of ((4-Fluoro-phenyl)-{2-[(1-phenyl-9H-β-carboline-3-carbonyl)-amino]-ethylamino}-methyl)-phosphonic acid dibutyl ester (**BCP-1**) in breast cancer cells (MCF-7 and MDA-MB-231) and in a non-tumor breast cell line (MCF-10A). **BCP-1** has an α-aminophosphonate unit linked to the β-carboline nucleus, and the literature indicates that compounds of these classes have high biological potential. In the present study, the mechanism of action of **BCP-1** was investigated through methods of spectrofluorimetry, flow cytometry, and protein expression analysis. It was found that **BCP-1** inhibited the proliferation of both cancer cell lines. Furthermore, it induced oxidative stress and cell cycle arrest in G2/M. Upregulation of apoptosis-related proteins such as Bax, cytochrome C, and caspases, as well as a decrease in the anti-apoptotic protein Bcl-2, indicated potential induction of apoptosis in the MDA-MB-231 cells. While in MCF-7 cells, **BCP-1** activated the autophagic death pathway, which was demonstrated by an increase in autophagic vacuoles and acidic organelles, in addition to increased expression of LC3I/LC3II and reduced SQSTM1/p62 expression. Further, **BCP-1** demonstrated antimetastatic potential by reducing MMP-9 expression and cell migration in both breast cancer cell lines. In conclusion, **BCP-1** is a promising candidate for breast cancer chemotherapy.

## 1. Introduction

Cancer is one of the main threats to human life and health [1], and of all types of cancer, breast cancer is one of the most prevalent malignant neoplasms worldwide, being recognized as the leading cause of mortality among women [2,3,4]. Current treatments include surgery, radiation therapy, hormonal therapy, immunotherapy, and targeted therapy; however, these treatments are not effective due to the frequent occurrence of adverse effects, systemic toxicity, chemoresistance, and tumor recurrence [3].

Therefore, there is an urgent need to provide new therapeutic approaches to achieve more effective, potent, and less toxic treatments [5].

Several biological properties, including the ability to inhibit the growth of tumor cells in vitro and in vivo have been attributed to various natural and/or synthetic products [6]. Compounds derived from β-carboline and α-aminophosphonate classes have attracted great attention from researchers due to the diverse therapeutic properties and biological potential widely reported [7,8].

The β-carbolines are alkaloids containing a tricyclic pyrido [3,4-*b*] indole ring in their structure. Compounds of this class have already been described in the literature as having a wide spectrum of pharmacological properties, including antitumor, antithrombotic, antiparasitic, antituberculosis, anticholinergic, and antimicrobial activities [9,10,11,12]. Cao [13] showed in their studies that a β-carboline alkaloid exhibited high toxicity against different tumor cells such as human hepatocarcinoma cells (HepG2) causing apoptosis through the mitochondrial signaling pathway. Furthermore, gastric cancer cells (MGC-803 and SGC-7901) treated with β-carboline derivatives induced autophagy and apoptosis triggered by Akt/mTOR/p70S6K and AMPK pathways [9]. Harmol, a β-carboline alkaloid induced autophagy in A549 lung cancer cells activated by the ERK1/2 pathway [12].

The α-aminophosphonates are among the most studied bioactive derivatives, which have been reported as having enzyme inhibitor, antiviral (including anti-HIV), antibacterial, antifungal, anticancer, and herbicidal activities [14,15,16,17,18]. The introduction of the α-aminophosphonate group in the core of synthetic compounds was also reported to increase their antitumor activity against lung cancer cells (NCI-H460), by inducing the intracellular reactive oxygen species (ROS) production, also affecting enzymes and genes related to cell death, in addition to generating cell cycle arrest in the G1 phase [18].

**Figure 1:**
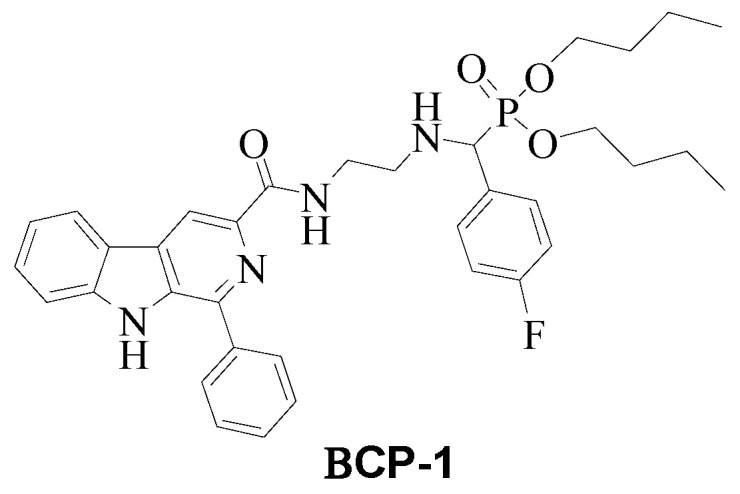
Structure of **BCP-1**.

Considering all the challenges in the treatment of breast cancer and the diverse biological properties already reported for β-carboline and α-aminophosphonate, in this study a compound containing both β-carboline and α-aminophosphonate moieties was designed giving rise to the unprecedented compound **BCP-1** ((4-Fluoro-phenyl)-{2-[(1-Phenyl-9*H*-β-carboline-3-carbonyl)-amino]-ethylamino}-methyl)-phosphonic acid dibutyl ester (Figure 1).

The objective of this work was to develop a compound capable of interfering with the proliferation of breast cancer cells (MCF-7 and MDA-MB-231) and to investigate its effect on different cell death pathways to determine the possible mechanism of action involved in the death of these cell lines. The study of intracellular proteins that act in apoptotic, necrotic, and autophagic signaling pathways, among other pathways, is important in the search for new compounds against cancer [19]. The signaling pathways related to apoptosis act in the inhibition of Bcl-2 proteins which makes the cell membrane permeable, leading to the activation of pro-apoptotic proteins. It generates the release of cytochrome C and the activation of the apoptotic cascade mechanism through caspases [20,21]. Furthermore, in preventing the proliferation of cancer cells, the autophagic signaling pathway can act through the activation of LC3I/LC3II proteins and inhibition of p62/SQSTM1 proteins [13,21].

Our results provided the first evidence that **BCP-1** contributes to breast cancer suppression in vitro, demonstrated by its action on the different cell death pathways depending on the cell line analyzed.

## 2. Results

### 2.1. Evaluation of the Cytotoxic Activity of **BCP-1** in Breast Cancer and Healthy Breast Cell Lines

The effect of the compound **BCP-1** was evaluated against two breast cancer cell lines, MCF-7 and MDA-MB-231, as well as a non-tumor breast cell line, MCF-10A, and the IC_50_ values obtained after 24 and 48 h of treatment are presented in Table 1. **BCP-1** treatment had both a time- and dose-dependent effect on the inhibition of breast cancer cell proliferation. The IC_50_ values in the MCF-10A cells were significantly higher than those found for the tumor cells, and thus, the selectivity index (SI) shows that the cytotoxic effects of **BCP-1** were selective for MCF-7 and MDA-MB-231 cells in relation to non-tumor MCF-10A cells.

The degree of selectivity of compounds can be expressed by their SI value, as suggested by Badisa [22] in which the degree of toxicity of the compound for the cancer cell line is compared to that in the normal cell line. SI is calculated as follows:

SI = IC_50_ normal cells/IC_50_ cancer cells

High SI values (>2) indicate selective toxicity towards the cancer cells, while an SI value of <2 shows general toxicity, that is to say, it causes cytotoxicity in both tumor and normal cells [22,23,24].

### 2.2. Morphological Changes Induced by **BCP-1** in Breast Cancer Cells

**Figure 2:**
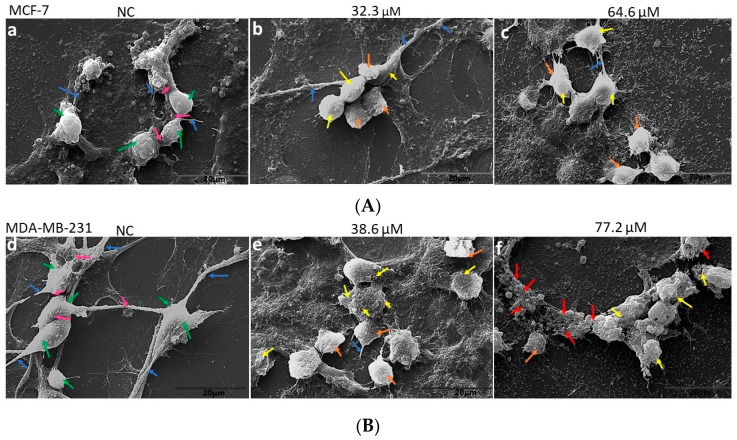
Evaluation of morphological changes in breast cancer cell lines following **BCP-1** treatment. (**A**) MCF-7 cells: (**a**) untreated negative control (NC), (**b**) treated with the IC_50_ of **BCP-1** (32.3 µM), (**c**) treated with the 2 × IC_50_ of **BCP-1** (64.6 µM). Green arrows indicate the intact body of healthy cells; blue arrows indicate microvilli; pink arrows indicate cell-cell junctions; yellow arrows indicate intact membrane without damage; orange arrows indicate cells with decreased cell volume, shrunken cells, and the absence of microvilli. (**B**) MDA-MB-231 cells: (**d**) NC, (**e**) treated with the IC_50_ of **BCP-1** (38.6 µM), (**f**) treated with the 2 × IC_50_ of **BCP-1** (77.2 µM). Green arrows indicate the intact body of healthy cells; blue arrows indicate microvilli; pink arrows indicate cell-cell junctions; yellow arrows indicate damaged membrane; red arrows indicate partial loss of cell membrane. Magnification: 5000×; scale bar: 20 μm.

The morphological changes in the breast cancer cells following **BCP-1** treatment were characterized by SEM. Untreated MCF-7 cells (NC) had intact membranes with microvilli (Figure 2A(a)), while MDA-MB-231 cells (NC) displayed abundant microvilli, as well as cell crowding, cell-to-cell connections, and elongated spindle features, all of which suggest healthy proliferation (Figure 2B(d)). After 48 h of treatment with the respective IC_50_ and 2 × IC_50_ concentrations of **BCP-1** (Table 1), both cell lines showed morphological changes. At both concentrations, MCF-7 cells showed a slight decrease in volume, with reduction of cell-cell junctions, but apparently the cell membrane remained intact (Figure 2A(b,c)). Meanwhile, **BCP-1**-treated MDA-MB-231 cells showed rounding, reduced microvilli, reduced volume, and cell membrane damage, suggesting cell death (Figure 2B(e,f)).

### 2.3. Effects of BCP-1 on ROS Production and GSH Levels in Breast Cancer Cells

**Figure 3:**
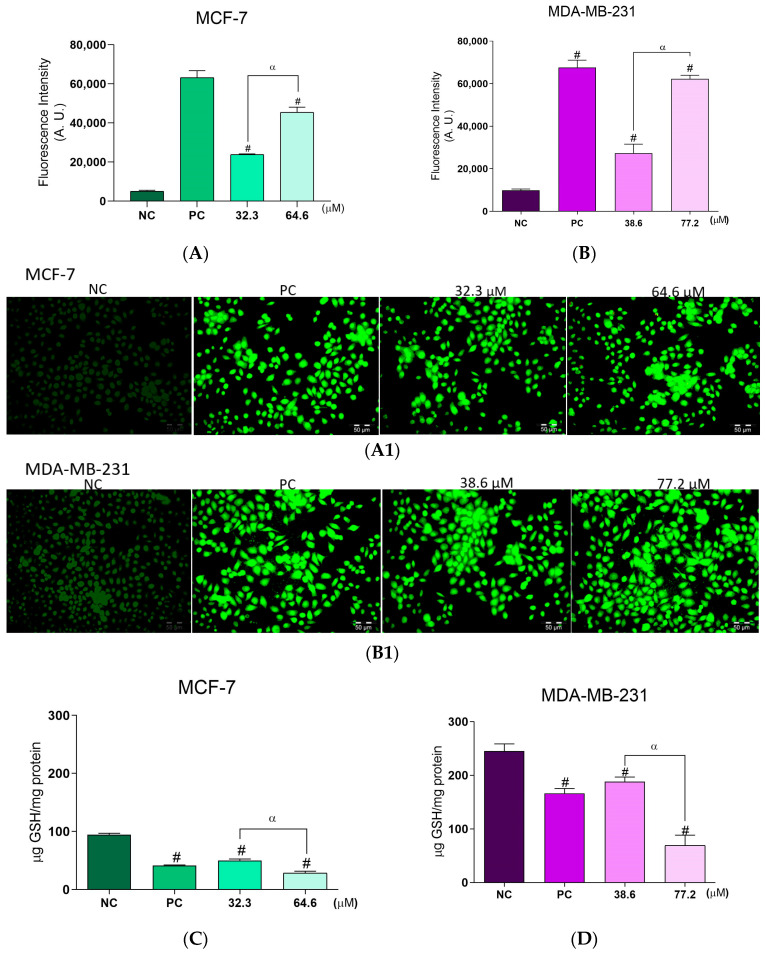
Evaluation of ROS production and GSH levels in breast cancer cells following **BCP-1** treatment. Cells were treated with the IC_50_ and 2 × IC_50_ (µM) of **BCP-1** for 48 h. H_2_O_2_ (200 µM) was used as the positive control (PC). (**A**,**B**) The cell-permeant indicator for ROS, H_2_DCFDA; (**A1**,**B1**) fluorescence images captured by microscopy; (**C**,**D**) GSH levels determined in the cell lysates. Fluorescence intensity was obtained by reading on a spectrofluorometer. Data are expressed as the mean ± SD of three independent experiments that were performed in triplicate. # *p* < 0.05, compared to the negative control (NC); α *p* < 0.05, between the IC_50_ and 2 × IC_50_ (one-way ANOVA followed by Tukey post-test). Scale bar: 50 μm.

Both breast cancer cell lines showed a significant increase in ROS production after **BCP-1** treatment. For MCF-7 cells, ROS production was increased by 368%, 793%, and 800% after treatment with the IC_50_ and 2 × IC_50_ of **BCP-1** and the PC (H_2_O_2_), respectively, relative to the level of the NC (Figure 3A). Similarly, MDA-MB-231 cells exhibited increases of approximately 180%, 530%, and 580% after treatment with **BCP-1** (IC_50_ and 2 × IC_50_) and the PC, respectively, relative to the NC (Figure 3B). Images obtained by fluorescence microscopy of the H_2_DCFDA marker corroborated the results obtained by the spectrofluorometric analysis, as it is possible to observe greater intensity of green fluorescence for both cell lines following **BCP-1** treatment (Figure 3(A1,B1)).

Considering the results on ROS production, the antioxidant defense system was also analyzed by assessing the GSH levels. **BCP-1** treatment of the two breast cancer cell lines suppressed the antioxidant defense system, as evidenced by the reduced levels of GSH compared to the NC. For MCF-7 cells treated with the IC_50_ and 2 × IC_50_ of **BCP-1** and the PC, the levels were reduced by approximately 48%, 70%, and 56%, respectively, relative to the NC (Figure 3C), while for MDA-MB-231 cells, the reductions were by 24%, 72%, and 32%, respectively (Figure 3D).

### 2.4. Effects of **BCP-1** on ΔΨm and Intracellular ATP Levels in Breast Cancer Cells

**Figure 4:**
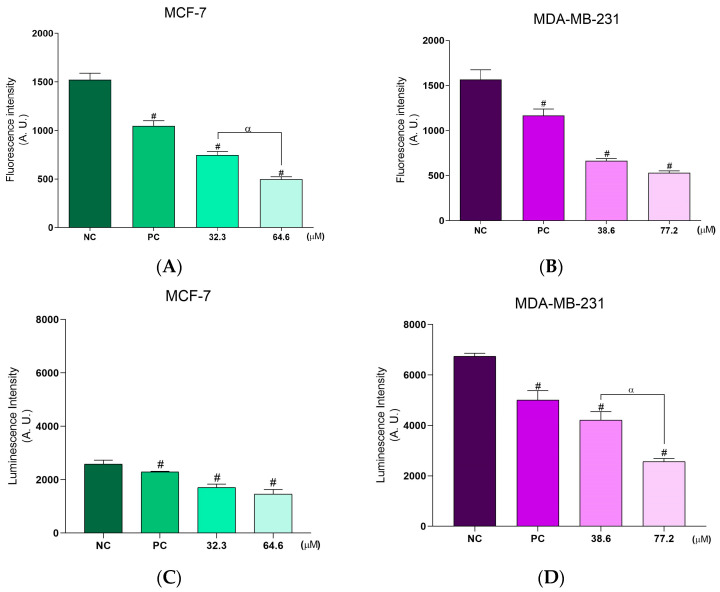
Assessment of ΔΨm and intracellular ATP levels in breast cancer cells following **BCP-1** treatment. Cells were treated with the IC_50_ and 2 × IC_50_ (µM) of **BCP-1** for 48 h. The positive controls (PC) were either (**A**,**B**) CCCP (100 µM) or (**C**,**D**) KCN (200 µM). (**A**,**B**) Fluorescence intensity of the cationic marker TMRE and (**C**,**D**) luminescence of the CellTiter-Glo^®^ reagent were determined in a microplate reader. Data are expressed as the mean ± SD of three independent experiments that were performed in triplicate. # *p* < 0.05, compared to the negative control (NC); α *p* < 0.05, between the IC_50_ and 2 × IC_50_ (one-way ANOVA followed by Tukey post-test).

**BCP-1** treatment of the two breast cancer cell lines demonstrated a significant dose-dependent reduction in ΔΨm. For MCF-7 cells treated with the IC_50_, 2 × IC_50_ of **BCP-1,** and the PC (CCCP), the ΔΨm was reduced by approximately 51%, 69%, and 33%, respectively, relative to the NC (Figure 4A), while for MDA-MB-231 cells, the reductions were by 58%, 66%, and 24%, respectively, relative to the NC (Figure 4B).

In terms of intracellular ATP levels, **BCP-1** treatment of the two breast cancer cell lines resulted in significant suppression of intracellular ATP production. Interestingly, this effect was more expressive in MDA-MB-231 cells. The ATP levels were reduced by approximately 34%, 43%, and 11%, in relation to the NC, for the MCF-7 cells treated with the IC_50_, 2 × IC_50_ of **BCP-1,** and the PC (KCN) (Figure 4C). For MDA-MB-231 cells, on the other hand, the ATP levels decreased by 38%, 62%, and 26%, respectively, in relation to the NC (Figure 4D).

### 2.5. Effect of BCP-1 on Lipid Peroxidation and Membrane Integrity in Breast Cancer Cells

**Figure 5:**
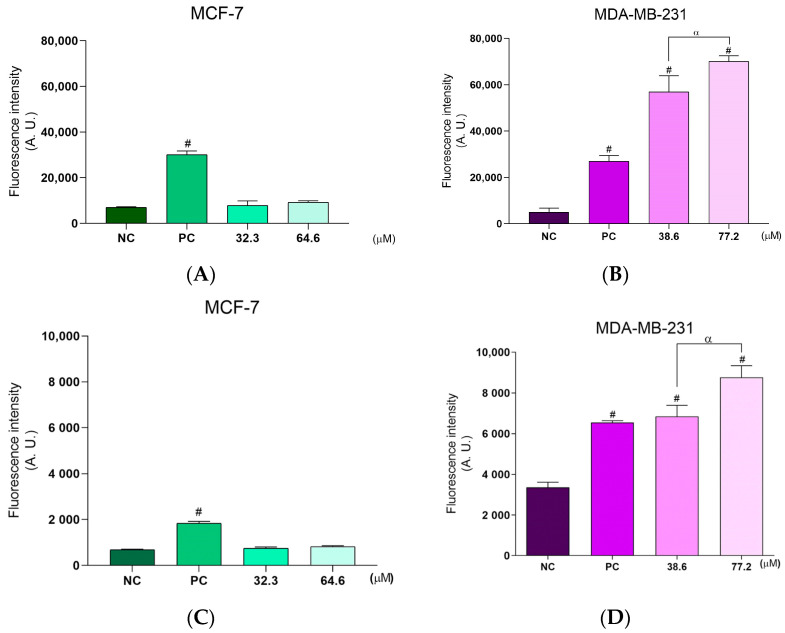
Evaluation of lipid peroxidation and membrane integrity in breast cancer cells following **BCP-1** treatment. Cells were treated with the IC_50_ and 2 × IC_50_ (µM) of **BCP-1** for 48 h. Lipid peroxidation (**A**,**B**) H_2_O_2_ (200 µM) and membrane integrity (**C**,**D**) digitonin (200 µM) were used as the positive controls (PC). Fluorescence intensity of (**A**,**B**) DPPP and (**C**,**D**) PI were determined in a microplate reader. Data are expressed as the mean ± SD of three independent experiments that were performed in triplicate. # *p* < 0.05, compared to the negative control (NC); α *p* < 0.05, between the IC_50_ and 2 × IC_50_ (one-way ANOVA followed by Tukey post-test).

MCF-7 cells did not show any changes in lipid peroxidation after treatment with both **BCP-1** concentrations used compared to the NC, while the PC (H_2_O_2_) caused a 400% increase (Figure 5A). Further, there were no changes in the membrane integrity of MCF-7 cells after treatment with the IC_50_ and 2 × IC_50_ of **BCP-1**, as there was no significant difference in the PI fluorescence intensity. The PC (digitonin), however, caused a 167% increase in PI fluorescence intensity compared to the NC (Figure 5C).

In contrast, MDA-MB-231 cells exhibited a significant increase in lipid peroxidation, by 800%, 1100%, and 380% after treatment with the IC_50_, 2 × IC_50_ of **BCP-1,** and the PC, respectively, when compared to the NC (Figure 5B). These cells further exhibited changes in cell membrane integrity, as there was an increase in PI fluorescence intensity of approximately 105%, 161%, and 95% after treatment with the IC_50_, 2 × IC_50_ of **BCP-1,** and the PC, respectively, when compared to the NC (Figure 5D).

### 2.6. Effect of **BCP-1** on the Cell Cycle Progression of Breast Cancer Cells

**Figure 6:**
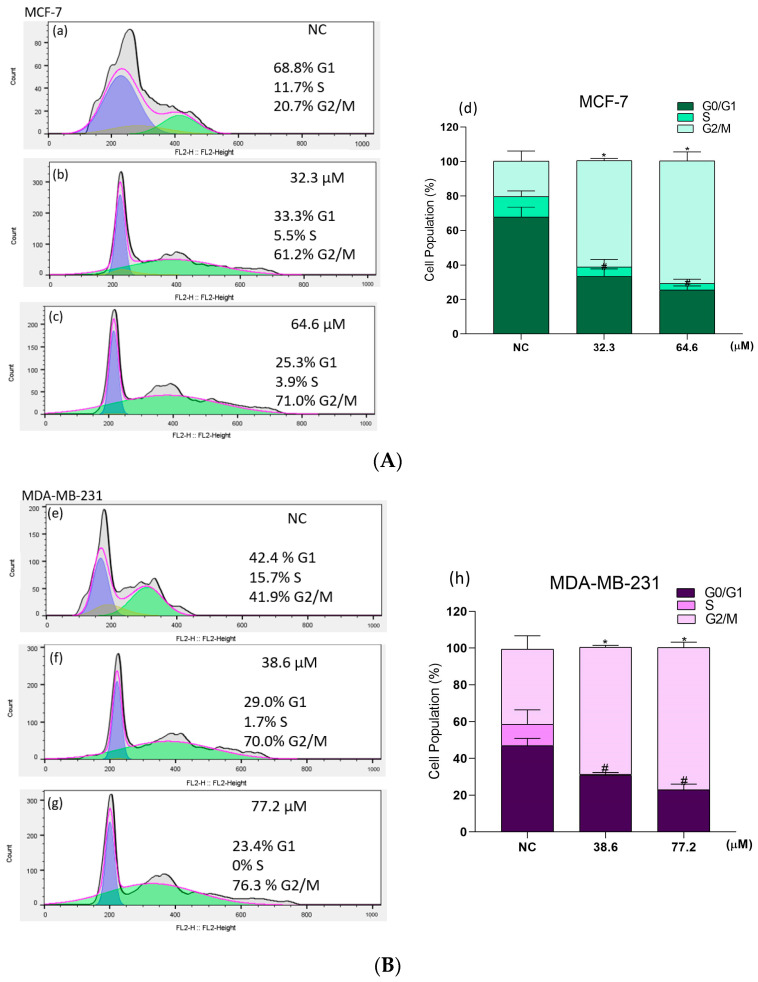
Cell cycle assessment of breast cancer cells following **BCP-1** treatment. Cells were treated with the IC_50_ and 2 × IC_50_ (μM) of **BCP-1** for 48 h. (**A**) MCF-7; (**B**) MDA-MB-231. (**a**,**e**) Negative control (NC); (**b**,**f**) cells treated with the IC_50_; (**c**,**g**) cells treated with 2 × IC_50_; (**d**,**h**) graphs to show the percentage of cells in each cell cycle phase. Data are expressed as the mean ± SD of three independent experiments that were performed in triplicate. # *p* < 0.05, compared to the G0/G1 phase of the NC; * *p* < 0.05, compared to the G2/M phase of the NC (one-way ANOVA followed by Tukey post-test). The lines are the mathematical calculations that the program makes to determine each of the phases. The dark gray part with the black line is the acquisition graph. The pink line is what the program used as the total DNA. Purple is what is in G0/G1, S phase yellow and G2/M green.

The different phases of the cell cycle of the breast cancer cells were quantified by flow cytometric analysis of PI-stained cells, which showed that treatment of both cell lines resulted in a significant accumulation of cells in the G2/M phase (Figure 6A,B). In the NC, approximately 68% of the MCF-7 cells were in G0/G1 phase, 11% were in S phase, and 21% were in G2/M phase (Figure 6A(a,d)). After **BCP-1** treatment with the IC_50_ and 2 × IC_50_, there was a reduction of 35% and 44% of cells in G0/G1, a decrease of 6% and 8% of cells in S phase, and an expressive increase in the percentage of cells in G2/M phase of 41% and 50%, respectively, in relation to the NC (Figure 6A(b–d)).

For the NC of MDA-MB-231 cells, 42% of cells were in G0/G1 phase, 15% were in S phase, and 41% were in G2/M phase (Figure 6B(e,h)). However, when cells were treated with the IC_50_ and 2 × IC_50_ of **BCP-1**, there was a reduction by 14% and 20% in the percentage of cells in G0/G1 phase compared to the NC. S phase showed no changes in the percentage of cells, but there was an increase of 28% and 35% of the percentage of cells in G2/M phase, in relation to the NC (Figure 6B(f–h)).

### 2.7. Effect of the **BCP-1** on DNA Fragmentation in Breast Cancer Cells

**Figure 7:**
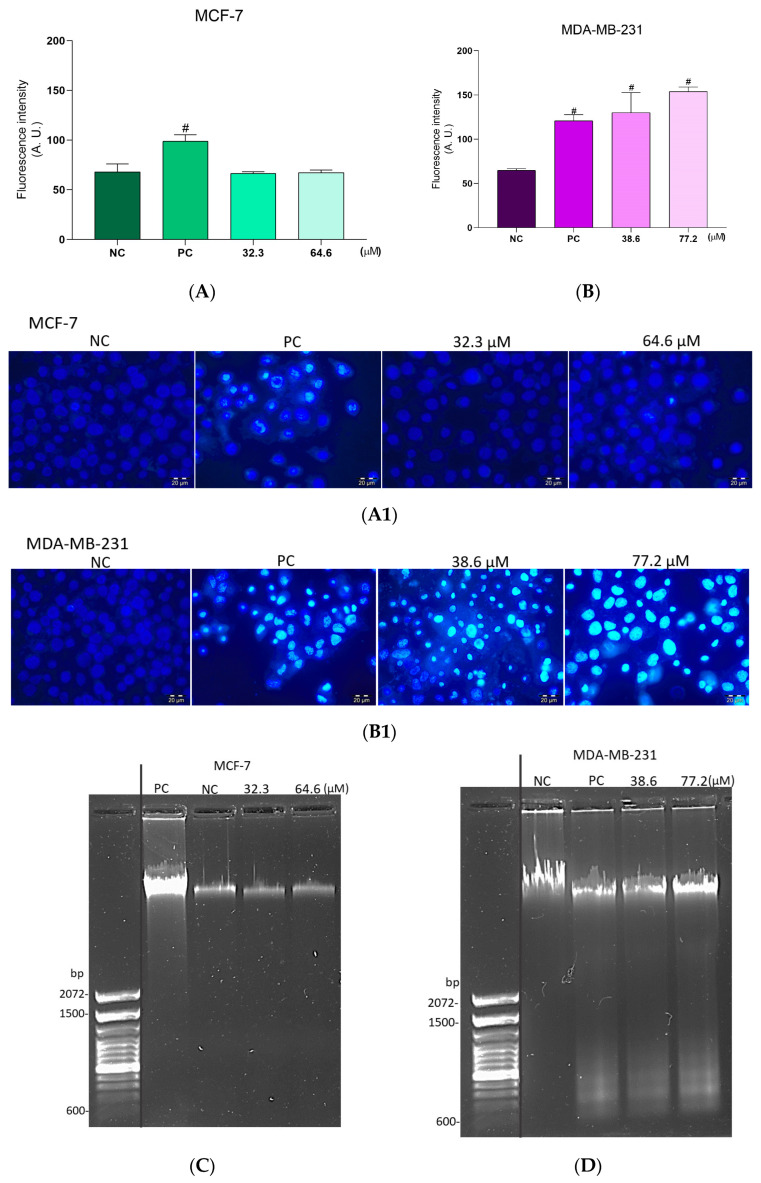
DNA integrity in breast cancer cells following BCP-1 treatment. Cells were treated with the IC_50_ and 2 × IC_50_ (µM) of BCP-1 for 48 h. CPT was used as a positive control (PC). Fluorescence of Hoescht 33342 was analyzed on a fluorescence microscope and the intensity was quantified using ImageJ. Scale bar: 50 μM. (**A**,**A1**) DNA integrity in MCF-7 cells. (**B**,**B1**) DNA integrity in MDA-MB-231 cells. Data are expressed as the mean ± SD of three independent experiments that were performed in triplicate. # *p* < 0.05, compared to the NC (one-way ANOVA followed by Tukey post-test). (**C**,**D**) DNA fragmentation was evaluated by agarose gel electrophoresis.

The integrity of the DNA in the breast cancer cells was also evaluated. MCF-7 cells, after the two **BCP-1** treatments, did not show an increase in the fluorescence intensity of the Hoechst 33342 dye, whereas the PC (CPT) showed a 44% increase in relation to the NC (Figure 7A,A1). To confirm this, MCF-7 cells were treated and submitted to electrophoresis in agarose gel, which showed that the PC had expressive DNA fragmentation, whereas cells treated with the two **BCP-1** concentrations showed no DNA fragmentation (Figure 7C).

In contrast, MDA-MB-231 cells showed an increase in Hoechst 33342 fluorescence intensity of 99%, 137%, and 86% after treatments with the IC_50_, 2 × IC_50_ of **BCP-1,** and the PC, respectively, relative to the NC, suggesting chromatin condensation (Figure 7B,B1). Results obtained by agarose gel electrophoresis also demonstrated intense fragmentation of the DNA in the treated cells when compared to the NC (Figure 7D).

### 2.8. Phosphatidylserine Exposure Analysis in Breast Cancer Cells Treated with **BCP-1**

**Figure 8:**
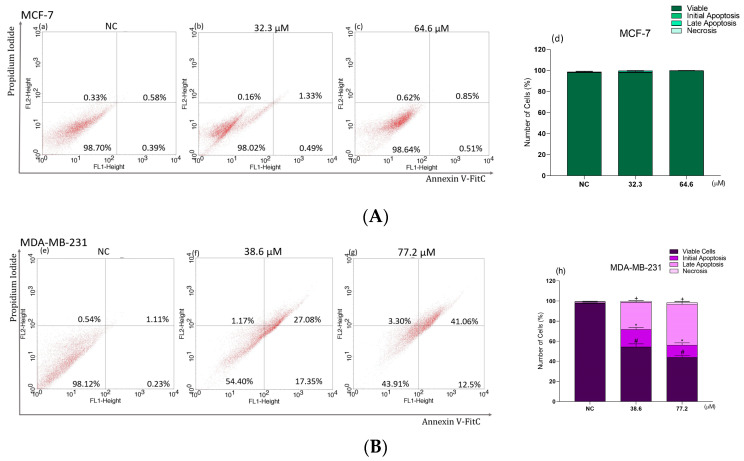
Determination of phosphatidylserine exposure in breast cancer cells following **BCP-1** treatment. Cells were treated with the IC_50_ and 2 × IC_50_ (μM) of **BCP-1** for 48 h. Cells were labeled with annexin V and PI and then assessed by flow cytometry. Data are expressed as the mean ± SD of three experiments that were performed in triplicate. (**A**) MCF-7: not significant; (**B**) MDA-MB-23: # *p* < 0.05, compared to the percentage of viable cells of the negative control (NC); * *p* < 0.05, compared to the percentage of cells in early apoptosis of the NC; + *p* < 0.05, compared to the percentage of cells in late apoptosis of the NC (one-way ANOVA followed by Tukey post-test).

Treatment with the IC_50_ and 2 × IC_50_ of **BCP-1** did not result in differences in the annexin V/PI staining of MCF-7 cells compared to the NC (Figure 8A(a–c)), suggesting that the death of these cells was possibly not occurring by apoptotic and/or necrotic pathways (Figure 8A(d)).

In contrast, treatment of MDA-MB-231 cells resulted in increases in the percentage of cells positive for annexin V alone and both annexin V and PI (Figure 8B(f,g)), in comparison to the NC (Figure 8B(e)). Cells labeled only with annexin V increased by approximately 17% and 12%, while cells with double labeling of annexin V and PI increased by 27% and 41% for the IC_50_ and 2 × IC_50_ treatments, respectively (Figure 8B(h)). Cells labeled with PI alone showed no significant increase in relation to the NC (Figure 8B(h)).

### 2.9. Effects of **BCP-1** on Cell Death and Expression of Apoptosis-Related Proteins in MDA-MB-231 Breast Cancer Cells

**Figure 9:**
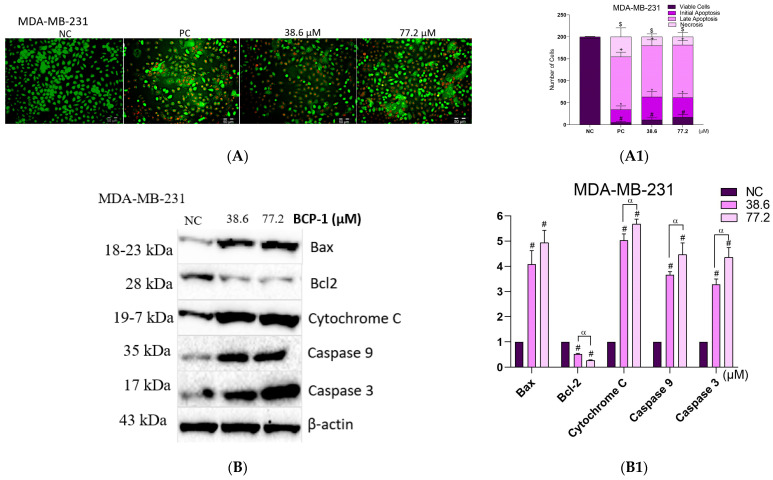
Analysis of cell death in MDA-MB-231 breast cancer cells following **BCP-1** treatment. MDA-MB-231 cells were treated with the IC_50_ and 2 × IC_50_ (μM) of **BCP-1** for 48 h. CPT (20 µM) was used as a positive control (PC). (**A**) Cells were double labeled with AO and PI, observed under fluorescence microscopy, and quantified by ImageJ. The images are representative of three independent experiments, which were performed in triplicate. (**A1**) # *p* < 0.05, compared with the number of viable cells in the negative control (NC); * *p* < 0.05, compared with the number of cells in early apoptosis in the NC; + *p* < 0.05, compared with the number of cells in late apoptosis in the NC; $ *p* < 0.05, compared with the number of cells in necrosis in the NC. (**B**) Expression of proteins assessed by Western blotting. (**B1**) Blots were quantified on a ChemiDoc^®^ XRS + Imaging System. # *p* < 0.05, compared to the expression of NC; α *p* < 0.05, between the IC_50_ and 2 × IC_50_ (one-way ANOVA followed by Tukey post-test).

As MDA-MB-231 cells exhibited characteristics of cell death via the apoptotic pathway, evaluation of apoptosis by AO and PI labeling and the expression of apoptosis-related proteins was performed. The labeling with AO/PI demonstrated that the percentage of viable cells after the two treatments with **BCP-1** and the PC (CPT) decreased by approximately 90% in relation to the NC. Cells in early apoptosis increased by approximately 1600%, 1380%, and 866% after treatment with the IC_50_, 2 × IC_50_ of BCP-1, and the PC, respectively, in relation to the NC. Further, increases in cells in late apoptosis of 3700%, 3900%, and 4000% were also observed following treatment with the IC_50_, 2 × IC_50_ of **BCP-1,** and the PC, respectively, in relation to the NC (Figure 9A,A1).

Additionally, Western blotting analysis revealed that expression levels of the Bax pro-apoptotic protein were up-regulated following **BCP-1** treatment. The expression was increased by 308% and 393% after treatment with the IC_50_ and 2 × IC_50_, respectively, relative to the NC (Figure 9B,B1). There was also a significant reduction in the expression of the anti-apoptotic protein Bcl-2 by 48% and 74% following treatment with the IC_50_ and 2 × IC_50_ of **BCP-1**, respectively (Figure 9B,B1). It was also possible to observe that cytochrome C expression increased by 403% and 467% after treatment with the IC_50_ and 2 × IC_50_ of **BCP-1**, respectively (Figure 9B,B1). Finally, caspase protein expression was investigated. After treatment with the IC_50_ and 2 × IC_50_ of **BCP-1**, increased expression of the initiator caspase-9 was observed by 226% and 446%, respectively, in relation to the NC (Figure 9B,B1). Observations were also made with respect to caspase-3, an executor protein in the process of apoptosis, the expression of which was increased by 229% and 335%, respectively (Figure 9B,B1).

### 2.10. Effects of **BCP-1** on the Number of Autophagic Vacuoles and Acidic Organelles, and the Expression of Proteins Related to Autophagy in MCF-7 Breast Cancer Cells

**Figure 10:**
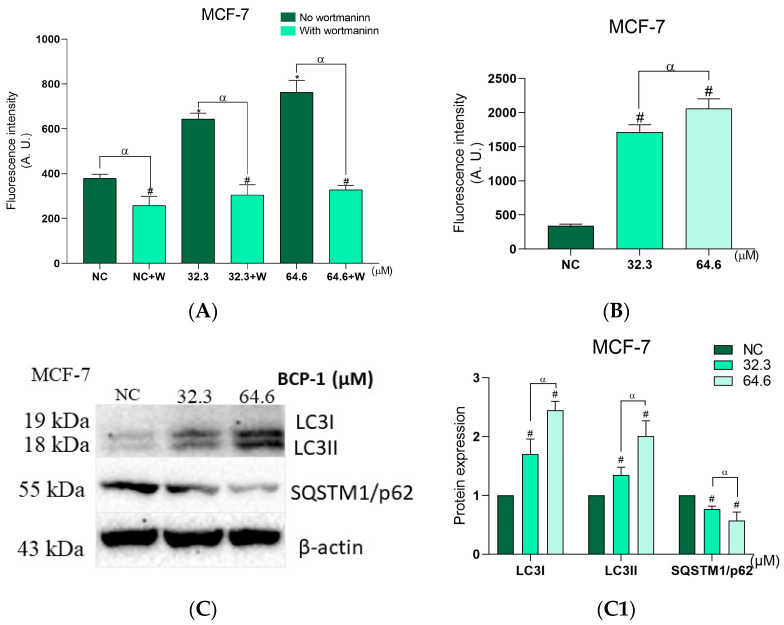
Analysis of cell death induced by **BCP-1** in MCF-7 breast cancer cells. MCF-7 cells were treated with the IC_50_ and 2 × IC_50_ (μM) of **BCP-1** for 48 h. Cells were stained with (**A**) MDC or (**B**) LysoTracker^®^ Red and then read in a microplate reader. Where indicated, cells were incubated with wortmannin (W) prior to **BCP-1** treatment. Data are expressed as the mean ± SD of three independent experiments that were performed in triplicate. (**A**) MCF-7: # *p* < 0.05, compared to the negative control (NC); * *p* < 0.05, compared to the NC; α *p* < 0.05, within the groups with and without wortmannin. (**B**) MCF-7: # *p* < 0.05, compared to the NC; α *p* < 0.05, between the IC_50_ and 2 × IC_50_. (**C**) Expression of proteins assessed by Western blotting. (**C1**) Blots were quantified on a ChemiDoc^®^ XRS + Imaging System. # *p* < 0.05, compared to the NC; α *p* < 0.05, between the IC_50_ and 2 × IC_50_ (one-way ANOVA followed by Tukey post-test).

As MCF-7 cells did not present features of cell death via the apoptotic pathway, and to determine the type of death triggered by **BCP-1** in these cells, we evaluated the number of autophagic vacuoles and acidic organelles, as well as the expression of proteins related to autophagy. The presence of autophagic vacuoles was analyzed using the MDC marker and MCF-7 cells after treatment with the IC_50_ and 2 × IC_50_ of **BCP-1** showed an increase in fluorescence intensity of 70% and 102%, respectively, in relation to the NC (Figure 10A). The autophagy inhibitor wortmannin was able to prevent the formation of autophagic vacuoles after **BCP-1** treatment, decreasing fluorescence intensity by 53% for the IC_50_ and 55% for 2 × IC_50_ compared to the respective treatments without wortmannin (Figure 10A). Furthermore, the NC with wortmannin showed a decrease in fluorescence intensity of 28% compared to the NC without wortmannin (Figure 10A).

The acidic organelles were evaluated by LysoTracker^®^ Red staining and the fluorescence intensity increased by 401% and 502% after treatment with the IC_50_ and 2 × IC_50_ of BCP-1, respectively, in relation to the NC (Figure 10B). Moreover, the Western blotting assay showed that the protein expression levels of LC3I and LC3II significantly increased by 70% and 34% after treatment with the IC_50_, and by 178% and 100% after treatment with 2 × IC_50_, respectively, compared to the NC (Figure 10C,C1). It was also verified that SQTM1/p62 protein expression levels were reduced by 24% and 43%, respectively, after treatment with the IC_50_ and 2 × IC_50_ of **BCP-1** compared to the NC (Figure 10C,C1). All these results indicated a possible induction of cell death via autophagy in this breast cancer cell line following **BCP-1** treatment.

### 2.11. Effects of the Antimetastatic Potential of BCP-1 on Breast Cancer Cells

**Figure 11:**
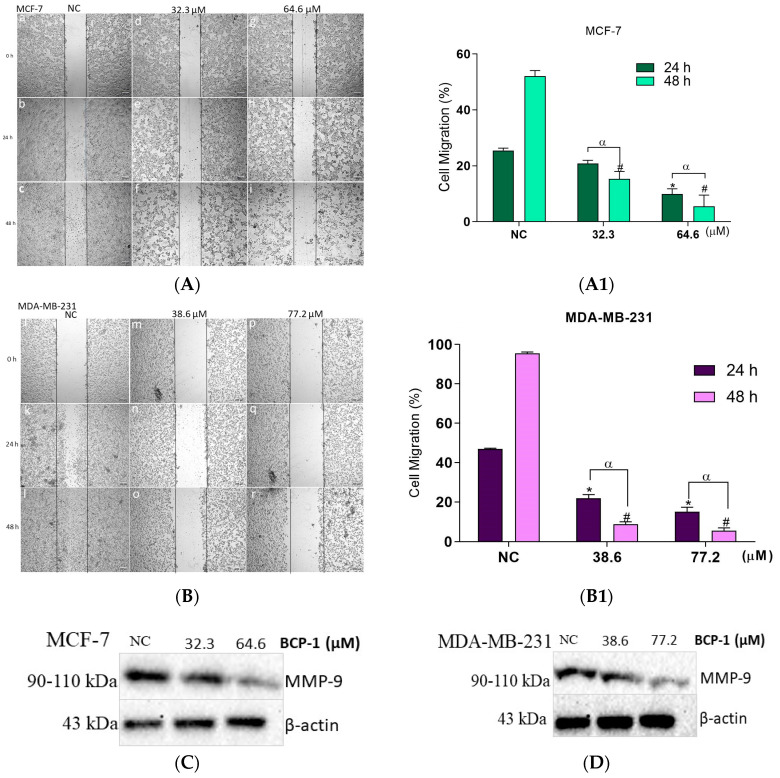
Evaluation of the antimetastatic potential of **BCP-1** on breast cancer cells. Cell monolayers were grown and then scraped. Cells were then treated with the IC_50_ and 2 × IC_50_ (μM) of **BCP-1** for 24 or 48 h and migration into the scraped area was assessed. (**A**,**B**) Images obtained using an inverted phase contrast microscope. Magnification: 4×; scale bar: 200 μM. (**A1**,**B1**) Data are expressed as the mean ± SD of three independent experiments that were performed in triplicate. * *p* < 0.05, compared to the NC at 24 h; # *p* < 0.05, compared to the NC at 48 h; α *p* < 0.05, between the incubation times of 24 and 48 h (two-way ANOVA followed by Tukey post-test). (**C**,**D**) Expression of MMP-9 protein by Western blotting. # *p* < 0.05, compared to the NC; α *p* < 0.05, between the IC_50_ and 2 × IC_50_ (one-way ANOVA followed by Tukey post-test). (**a**–**i**) represent the result of the migration assay of MCF-7 cells. (**j**–**r**) represent the result of the migration assay of MDA-MB-231 cells.

Using a cell migration assay, it was possible to observe that non-treated MCF-7 cells showed a migration into the scratched area of approximately 25% and 53% after 24 and 48 h, respectively, indicating closure of more than half of the total scratched area in the longest time at 48 h (Figure 11A(a–c)). After treatment with the IC_50_ of **BCP-1**, cell migration at 24 and 48 h decreased to approximately 20% and 14%, respectively, (Figure 11A(e,f) in relation to the NC. Regarding the treatment with 2 × IC_50_, the cell migration at 24 and 48 h was approximately 9% and 5% (Figure 11A(h,i)), as represented in the graph (Figure 11A1). Further, non-treated MDA-MB-231 cells exhibited a migration of approximately 47% and 95% after 24 and 48 h, respectively, indicating almost complete closure of the total scratched area (Figure 11B(j–l)). In treatments with the IC_50_, cell migration was reduced to approximately 22% and 8%, at 24 and 48 h, respectively, (Figure 11B(n,o)) in relation to the NC, while the treatments with 2 × IC_50_ led to approximately 14% and 5% of cell migration after 24 and 48 h, respectively (Figure 11B(q,r),B1).

As **BCP-1** was effective in inhibiting migration in both cell lines, we also evaluated the expression of the matrix metallopeptidase (MMP)-9 protein. After treatment of MCF-7 cells with the IC_50_ and 2 × IC_50_ of **BCP-1**, MMP-9 expression levels significantly decreased by 26% and 60%, respectively, relative to the NC (Figure 11C). MDA-MB-231 cells also exhibited a decrease in the expression of this protein by approximately 31% and 61%, respectively, after treatment with the IC_50_ and 2 × IC_50_ compared to the NC (Figure 11D). Taken together, these results suggest that **BCP-1** has inhibitory effects on the migration of these cells.

### 2.12. Effects of **BCP-1** on Breast Cancer Cell Colony Formation

**Figure 12:**
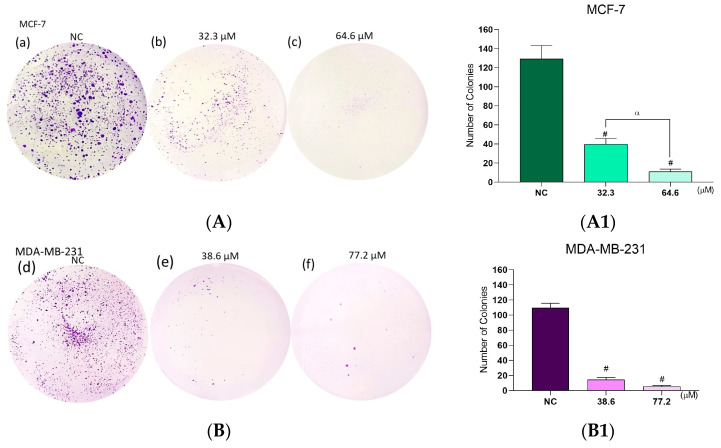
Assessment of colony formation in a breast cancer cell line following **BCP-1** treatment. Cells were treated with the IC_50_ and 2 × IC_50_ (μM) of **BCP-1** for 48 h and then submitted to the clonogenic assay. Colonies were stained with crystal violet and counted. (**A**) MCF-7; (**B**) MDA-MB-231. Data are expressed as the mean ± SD of three independent experiments that were performed in triplicate. (**A1**,**B1**) # *p* < 0.05, compared to the NC; α *p* < 0.05, between the IC_50_ and 2 × IC_50_ (one-way ANOVA followed by Tukey post-test).

In the clonogenic assay, untreated MCF-7 cells (NC) showed approximately one hundred and forty-seven colonies of cells (Figure 12A(a)), and after treatment with the IC_50_ and 2 × IC_50_ of **BCP-1,** a decrease in the number of colonies was observed, to only forty-one and eleven colonies, respectively, (Figure 12A(b,c)), representing a reduction of 71% and 92%, in relation to the NC (Figure 12A1). Meanwhile, there were one hundred and nine colonies in the NC of MDA-MB-231 cells (Figure 12B(d)), and when treated with the IC_50_ and 2 × IC_50_, a reduction to fifteen and six colonies was observed, respectively (Figure 12B(e,f)), representing a decrease of approximately 86% and 94% compared to the NC (Figure 12B1).

## 3. Discussion

Considering the limitations in the current treatments of breast cancer, there is a need for new actives and therapeutic approaches that act more specifically on tumor cells to eliminate and/or reduce the chances of recurrence and adverse effects [25]. In this context, considering the potent cytotoxic effects and the molecular mechanisms already reported for β-carboline and α-aminophosphonate derivatives against different types of cancer [12,26], the synthetic compound **BCP-1**, containing both the β-carboline and α-aminophosphonate moieties, was evaluated in MCF-7 and MDA-MB-231 breast cancer cells.

**BCP-1** was able to reduce tumor cell viability in a time- and dose-dependent manner. In contrast, healthy breast epithelial cells were not significantly affected, resulting in a SI >3 after 48 h of treatment. SI values >2 are considered significantly selective towards one target, in this case, towards the cancer cells [22,23]. Yeh [27] treated MCF-7 and MDA-MB-231 cells with flavopereirine, a β-carboline alkaloid derivative, and found a reduction in cell viability, demonstrating the cytotoxic potential of this group of compounds. From SEM analysis, morphological changes were observed in MCF-7 and MDA-MB-231 cells, confirming the cytotoxic potential of **BCP-1**. Similarly, Razak [28] showed that MCF-7 and MDA-MB-231 cells treated with the flavonoid-derived compound eupatorin also exhibited similar changes in morphology and cell membrane destruction, evidencing the cytotoxic effect of the tested compound.

ROS are important signaling molecules in various cellular processes such as growth, differentiation, and death. They are normally produced during mitochondrial cellular respiration and energy generation, and then degraded by the antioxidant defense system [29]. Excess ROS can cause damage, and thus, for normal cell functioning there must be a compensation between ROS formation and levels of antioxidant defenses [30]. When there is an imbalance between levels of antioxidant defense and ROS production, oxidative stress occurs, which can mediate the induction of different cell death pathways [18]. In our study, it was verified that after **BCP-1** treatment, both tumor cells exhibited excessive ROS production, in addition to a reduction in the levels of antioxidant defense, as demonstrated by the lower GSH levels, leading to redox imbalance in the cells. According to Zhang [21], a β-carboline derivative also promoted an increase in ROS production and a decrease in GSH levels in gastric carcinoma and colorectal cancer cells, leading to cell death.

Mitochondria are one target of ROS and can contribute to mitochondrial damage, such as the reduction of △Ψm and consequent ATP production [18]. When these ROS levels are not kept stable, it causes changes in the functioning of cell physiological activities [31,32]. In the present study, it was observed that **BCP-1**-treated MCF-7 and MDA-MB-231 cells had reduced △Ψm, which possibly led to a decrease in intracellular ATP levels.

The plasma membrane is formed mainly by lipids and proteins that guarantee the structure of the cell membrane [33]. Lipid peroxidation is one of the main causes of the impairment of cell membranes, being related to functional and structural integrity, and may induce changes in their permeability [34]. The occurrence of lipid peroxidation was verified in MDA-MB-231 cells treated with **BCP-1**, which possibly led to changes in their membrane structures. Moreover, an increase in PI fluorescence intensity was observed, confirming the loss of membrane integrity. In contrast, MCF-7 cells did not show cell membrane damage as they did not exhibit lipid peroxidation or increased PI fluorescence intensity.

The cell cycle comprises a set of phases that cells go through to duplicate themselves, giving rise to new cells. It is known that cycle arrest is one of the main mechanisms of cell growth inhibition, and this regulation is a key method to control tumor spread [35]. Our results showed that after treatment with **BCP-1**, the cell cycle of both MCF-7 and MDA-MB-231 cells was interrupted in the G2/M phase. The G2/M phase is responsible for the synthesis of RNA and proteins, and the checkpoint in this phase prevents the entry of the cell into mitosis, as it is the interval between DNA duplication and the beginning of cell division [36]. Recent studies have also shown that cancer cells treated with α-aminophosphonate and β-carboline derivatives induced cycle arrest in the G2/M phase, relating this event to the control of tumor propagation [9,35].

Considering that cell DNA damage is an important indicator of apoptosis [37], chromatin condensation and DNA fragmentation were analyzed in the breast cancer cells by staining with Hoechst-33342 and assessment by agarose gel electrophoresis. MDA-MB-231 cells treated with **BCP-1** exhibited chromatin condensation and apparent DNA fragmentation. Differently, MCF-7 cells had intact DNA and compact chromatin after treatment. Studies by Sarkar [38] and Ye [18] also demonstrated fragmented DNA and chromatin condensation of liver cancer (HepG2) and lung cancer (NCI-H460) cells after treatment with derivative β-carboline and α-aminophosphonate, respectively, and related this result to the induction of cell death.

To analyze the possible occurrence of apoptosis in cells, assays involving double staining with either annexin V/PI or AO/PI were performed. Most treated MDA-MB-231 cells were labeled with both annexin V and PI, in addition, to being double labeled with AO/PI, confirming that **BCP-1** may induce apoptosis in this cell lineage, which is supported by the other results that demonstrated striking features of apoptosis. Interestingly, MCF-7 cells did not show annexin V/PI or AO/PI double labeling, indicating that the death pathway of this lineage is possibly not related to apoptosis. Supporting our data, Yeh [27] showed that flavopereirine also exhibited a remarkable effect in inducing apoptosis of MDA-MB-231 cells, determined by annexin V/PI double labeling.

According to Cao [20], mitochondria play an essential role in apoptosis by triggering mitochondrial membrane permeabilization. Mitochondria-mediated apoptosis is regulated by proteins of the Bcl-2 family, which are divided into two subfamilies: anti-apoptotic Bcl-2 and pro-apoptotic Bax. Therefore, the balance of these proteins is vital. Caspases belong to the protease family and are essential for carrying out apoptosis. Caspase-9 is one of the initiating caspases in the intrinsic apoptosis pathway and its activation requires the release of cytochrome C from mitochondria into the cytosol [31]. Caspase-3 activation correlates with caspase-9 activation, and during the process of apoptosis, caspase-3 plays a key role in executing this cellular death pathway [20]. In our study, it was verified whether the apoptotic pathway induced by **BCP-1** in MDA-MB-231 cells was related to the mitochondria. There was a positive regulation in Bax expression and a negative regulation of Bcl-2 expression, and this imbalance possibly led to the release of cytochrome C and the consequent activation of caspases. This cascade of events was likely responsible for the execution of apoptosis in these cells.

According to a study by Gozuacik [39], the inhibition of apoptosis can lead to cell death by autophagy, and the inhibition of autophagy can trigger apoptosis. Although the role of autophagy in cancer is still controversial, as it can protect cancer cells from apoptosis and metastasis, but can also induce cell death, prevent metastasis, and even increase chemosensitivity [40]. Lou [41] reported that autophagy plays a critical role in controlling cell proliferation, differentiation, and death.

Therefore, considering the previous data obtained for MCF-7 cells, which did not show a pattern of apoptotic cell death, the possible occurrence of autophagy was considered and the formation of autophagosomes was analyzed by MDC marker. This marker is specific for autophagic vacuoles and, in **BCP-1**-treated cells, a significant increase of autophagic vacuoles was observed. These data were further confirmed using wortmannin, an autophagy inhibitor, that was able to reduce autophagic vacuoles of treated cells. Zhang [14] also reported that the induction of autophagy was significantly reduced in MCF-7 cells by using the same inhibitor. Furthermore, our data are also supported by other studies on autophagic cell death induced β-carboline derivatives in A549 lung cancer cells [12].

To confirm that autophagy was induced by **BCP-1** in MCF-7 cells, an assay was performed with the LysoTracker^®^ marker and the expression of some proteins related to autophagy were evaluated by Western blotting analysis. LysoTracker^®^ allows the identification of lysosomes, which play an important role in autophagy, since they help in the degradation mechanism of cytoplasmic components [42]. The LC3I protein, one of the key factors in the development of the autophagic membrane, is currently an autophagosome marker, and the LC3II protein reflects the structures related to autophagy [43]. Monitoring LC3 levels is a standard method to assess autophagic flux [44].

Furthermore, the expression of the p62/SQSTM1 protein was evaluated; this is an intracellular protein located in the cytoplasm, nucleus, autophagosomes, and lysosomes in tissues [45], and it is a ubiquitin-binding protein involved in cell signaling, oxidative stress, and autophagy. To induce autophagy, p62/SQSTM1 forms oligomers that interact with LC3-tagged proteins leading to the initiation of autophagosome formation [46]. p62/SQSTM1 is not only an autophagy receptor, but also an autophagy substrate, as it is taken up by the autophagosome and degraded by the autophagolysosome [43]. p62/SQSTM1 and LC3 are used together as markers of autophagy in cancer research [13].

In our study, **BCP-1** treated MCF-7 cells showed a significant increase in the presence of lysosomes, as identified by the increase in the fluorescence intensity of LysoTracker^®^, in addition to increased expression of LC3I/LC3II proteins. In contrast, there was a significant reduction in p62/SQSTM1 expression. Thus, the data suggest that the mechanisms involved in cell death triggered by **BCP-1** in MCF-7 cells may be related to autophagy. Supporting our findings, [31] demonstrated that after treatment of melanoma cells (B16) with a compound called harmine derivative β-carboline, there was a positive regulation of LC3II expression. Abe reported in his study that after treatment with harmol, a β-carboline derivative, human glioma cells (U251MG) were induced to autophagy and apoptotic cell death [47]. In addition, Tang [48] also reported that autophagosome formation was induced by curcumin with increased LC3 expression and p62/SQSTM1 degeneration in lung cancer cells (A549), and the authors correlated these data to the induction of autophagy in the cells.

Another important factor in cancer is the process of metastasis. Metastasis is the leading cause of death in cancer patients, and it is characterized by a multi-step process that promotes the migration of cancer cells to distant organs. Several proteins are involved in this process, such as MMP-9, which plays an important role in promoting tumor growth [49]. Concerning breast cancer, metastases are of great relevance, especially in patients with the triple negative breast cancer subtype (TNBC) who are at high risk of spreading [50]. Given that, the effect of **BCP-1** on cell migration was evaluated in both MCF-7 and MDA-MB-231 cells and it was possible to observe that the migration rate was significantly reduced. Analysis of MMP-9 protein expression confirmed the possible antimetastatic potential of **BCP-1,** since a significant decrease in MMP-9 expression was observed for both strains. According to Zhu [51], harmine was able to inhibit cell migration by reducing MMP-2 and MMP-9 expression levels in glioblastoma, and Gao [49] reported that harmine inhibited MMP-9 expression in ovarian cancer cells. Likewise, nasopharyngeal carcinoma cells (NPC-039) also showed inhibition of cell migration when treated with an α-aminophosphonate derivative [52].

Finally, we evaluated the effect of **BCP-1** on the ability to inhibit the survival of MCF-7 and MDA-MB-231 cells through the clonogenic assay. This test is based on the ability of cells to adhere and form colonies, since adhesion represents one of the first and main phases for the formation of colonies [53]. **BCP-1** inhibited colony formation in both breast cancer cells, which may be correlated with the reduced viability. Zhang [21] showed that after treatment with β-carboline derivatives, hepatocellular carcinoma cells (HepG2) and HCT-116 had significantly decreased colony formation, relating this data to the cytotoxic effect of the compound.

In summary, **BCP-1** induced several alterations in both breast cancer cell lines mediated by oxidative stress, depolarization of the mitochondrial membrane potential with consequent reduction in intracellular ATP levels, and all alterations possibly led to the triggering of cell death in both cell lines. **BCP-1** activated the apoptotic death pathway in MDA-MB-231 cells demonstrated by annexin V/PI labeling, AO/PI labeling, chromatin condensation, and DNA fragmentation, and these data were confirmed by the regulation of proteins related to apoptosis. Differently, **BCP-1** possibly induced the autophagic death pathway in MCF-7 cells, as evidenced by an increase in autophagic vacuoles and acidic organelles, in addition to the regulation of the expression of key proteins in the development of autophagy.

Considering all the changes observed in vitro in the breast cancer cells after treatment with **BCP-1**, the present study has demonstrated that this compound has promise as a chemotherapeutic candidate. Therefore, future studies on the in vivo efficacy, pharmacokinetics, and toxicity of the compound are needed to better understand its antitumor effect and mechanism of action.

## 4. Material and Methods

### 4.1. Reagents

Secondary mouse IgGκ BP-HRP antibody (sc-516102), mouse anti-Bax (sc-20067), mouse anti-Bcl-2 (sc-7382), mouse anti-caspase-9 (sc-56076), mouse anti-caspase-3 (sc-56053), mouse anti-MMP-9 (sc-93859), mouse anti-cytochrome C (sc-13561), mouse anti-LC3I/LC3II (sc-271625), mouse anti-p62/SQSTM1 (sc-28359), and mouse anti-β-actin (sc-69879) were purchased from Santa Cruz Biotechnology ((Santa Cruz, CA, USA). Dulbecco’s Modified Eagle Medium (DMEM) and Fetal Bovine Serum (SFB) were purchased from Gibco (GRAND Island, NE, USA). Bradford reagent was obtained from Bio-Rad. CellTiter-Glo^®^ was purchased from Promega (WI, USA). Annexin V-FITC, camptothecin (CPT), dichlorodihydrofluorescein diacetate (H_2_DCFDA), Hoechst 33342, 3-(4,5-dimethylthiazol-2-yl)-2,5-diphenyltetrazolium bromide (MTT), diphenyl-1-pyrenylphosphine (DPPP) DNase-free RNase, and LysoTracker^®^ Red were purchased from Thermo Fisher Scientific (CA, USA). Carbonyl cyanide m-chlorophenylhydrazone (CCCP), glutathione (GSH), acridine orange (AO), tetramethylrhodamine ethyl ester (TMRE), propidium iodide (PI), o-phthaldialdehyde (OPA), and monodansylcadaverine (MDC) were purchased from Sigma-Aldrich (MO, USA).

### 4.2. Synthesis and Characterization of β-Carboline-α-Aminophosphonate BCP-1

**Figure 13:**
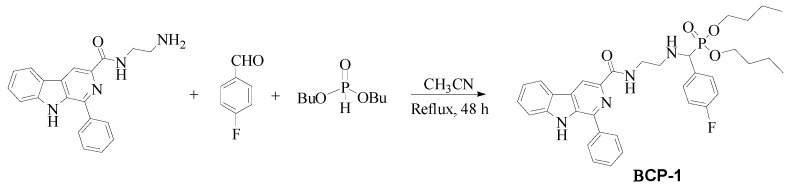
Synthesis of the β-carboline-α-aminophosphonate **BCP-1**.

The compound ((4-Fluoro-phenyl)-{2-[(1-phenyl-9*H*-β-carboline-3-carbonyl)-amino]-ethylamino}-methyl)-phosphonic acid dibutyl ester, named in this study as **BCP-1**, was synthesized from the reaction of *N*-(2-aminoethyl)-1-(phenyl)-β-carboline-3-carboxamide with 4-fluorobenzaldehyde and dibutyl phosphite (Figure 13), at the Chemistry Laboratory of the Maringá State University, according to procedure described by Oliveira [54]. A suspension of the intermediate *N*-(2-aminoethyl)-1-(phenyl)-β-carboline-3-carboxamide (0.330 g; 1.0 mmol) was prepared in acetonitrile (50 mL) as described by Baréa [55], to which 4-fluorobenzaldehyde (1.0 mmol) and dibutylphosphite (1.5 mmol) were added. The reaction mixture was refluxed for 48 h, and then the solvent was evaporated under vacuum. The amorphous solid formed was purified by chromatographic column (silica gel 60, 0.063–0.200 mm; EtOAc: MeOH, 1:1) to yield the compound **BCP-1** (60% yield, Molecular mass (MM) of 630.28 g/mol, soluble in DMSO).

Compound **BCP-1** was characterized with the basis on their ^1^H and ^13^C NMR data. ^1^H NMR (300 MHz, CDCl_3_) δ ppm: 8.77 (s, H-4), 8.16 (d, *J* = 7.8 Hz, H-5), 7.36 (m, H-6), 7.20–7.49 (m, H-7, H-8), 9.21 (s, 9-NH), 8.01 (d, *J* = 7.5 Hz, H-2′/6′), 7.49–7.62 (m, H-3′/5′, H-4′), 9.08 (t, H = 6.0 Hz, NH-C=O), 7.49–7.62 (m, H-2′′/H-6′′), 6.95 (t, *J* = 8.8 Hz, H-3′′/5′′), 4.36 (d, J_HP_ = 18.0 Hz, H-CP), 3.62 (m, CH_2_-NHC=O), 3.10 (m, -CH_2_NH), 3.75–4.10 (m, -CH_2_O), 1.27–1.72 (m, OCH_2_CH_2_CH_2_-), 0.63–0.96 (m, CH_3_). ^13^C NMR (75 MHz, CDCl_3_) δ ppm: 141.1 (C-1), 140.0 (C-3), 113.7 (C-4), 130.7 (C-4a), 122.2 (C-4b), 122.3 (C-5), 121.1 (C-6), 129.4 (C-7), 112.2 (C-8), 141.2 (C-8a), 134.8 (C-9a), 137.9 (C-1′), 122.3 (C-2′/6′), 129.3 (C-3′/5′), 128.5 (C-4′), 134.9 (C-1′′), 130.1 (C-2′′/6′′), 131.3 (C-3′′/5′′), 160.0 (C-4′′), 166.7 (C=O), 37.9 (CH_2_-NHC=O), 47.5 (-CH_2_NH), 58.7 (d, ^1^J_CP_ = 151.5 Hz, H-CP), 65.7, 65.8 (d, ^2^J_CP_ = 7.5 Hz, -CH_2_O), 32.5, 32.6 (OCH_2_CH_2_-), 18.7, 18.9-(OCH_2_CH_2_CH_2_-), 13.7, 13.8 (CH_3_).

### 4.3. Cell Lines and Cell Culture

The cells MDA-MB-231 and MCF-10A were kindly provided by Dr. José Andrés Morgado Diaz (Researcher, Instituto Nacional do Câncer (INCA), Rio de Janeiro, Brazil) and the MCF-7 cells were purchased from the American Type Culture Collection (ATCC). MCF-7 and MDA-MB-231 breast cancer cells were cultivated in culture flasks containing Dulbecco’s Modified Eagle Medium (DMEM) supplemented with 10% fetal bovine serum (FBS), 2 mM l-glutamine, and 0.5 U/mL penicillin/streptomycin. The healthy breast epithelial cell line, MCF-10A, was cultured in DMEM supplemented with 2.5 mM l-glutamine, 5% horse serum, 10 µg/mL human insulin, 0.5 µg/mL hydrocortisone, and 10 ng/mL epidermal growth factor (EGF). The cells were maintained at 37 °C in an atmosphere of 5% CO_2_.

In all experiments, cell suspensions were obtained following enzymatic treatment of the cell monolayer (0.2 g/100 mL trypsin, 0.02 g/100 mL EDTA), and the cell concentration of the suspension was adjusted to 2.5 × 10^5^ cells/mL and then plated in the appropriate microplate depending on the assay.

### 4.4. Evaluation of Cell Viability

Cell viability was determined by the colorimetric MTT assay. MCF-7, MDA-MB-231, and MCF-10A cells were plated in 96-well plates and maintained at 37 °C in an atmosphere of 5% CO_2_ for 24 h. The cells were treated with different concentrations of **BCP-1** (2–150 µM) for 24 and 48 h. After each exposure period, a solution containing MTT (2 mg/mL) was added in each well, followed by incubation at 37 °C for 4 h. The absorbance was then read in a microplate reader (BioTek PowerWave XS; Winooski, VT, USA) at 570 nm and the analysis was performed by non-linear regression. The inhibitory concentration for 50% of the cells (IC_50_) was determined as the concentration capable of reducing 50% of the optical density of the treated cells relative to the untreated negative control (NC).

For all the subsequent experiments, cultured breast cancer cells were treated with the respective IC_50_ and 2 × IC_50_ concentrations of **BCP-1** and maintained at 37 °C in an atmosphere of 5% CO_2_ for 48 h (unless stated otherwise): 32.3 µM and 64.6 µM for MCF-7 cells, and 38.6 µM and 77.2 µM for MDA-MB-231 cells.

### 4.5. Evaluation of the Cell Morphology by Scanning Electron Microscopy (SEM)

For morphological analysis, MCF-7 and MDA-MB-231 cells were plated on glass coverslips in 24-well plates. Cells were then treated with **BCP-1** for 48 h, then fixed in 2.5% glutaraldehyde in 0.1 M phosphate buffer (pH 7.3) for 90 min. Samples were then dehydrated in an increasing concentration series of ethanol (30–100%), subjected to the critical point, metallized, and analyzed under SEM (FEI Quanta 250; FEI Company, OR, USA).

### 4.6. Evaluation of the Production of Reactive Oxygen Species (ROS)

MCF-7 and MDA-MB-231 cells plated in 96-well plates were treated with **BCP-1**. H_2_O_2_ (200 µM) was used as a positive control (PC). Cells were then labeled with H_2_DCFDA (10 μM) at 37 °C for 45 min in the dark. Fluorescence was quantified in a microplate reader (VICTOR™ X3; PerkinElmer, Waltham, MA, USA) with excitation/emission wavelengths of 485/535 nm, respectively. The fluorescence intensity of all experiments was normalized by counting the number of cells, as according to Souza [56]. The ROS accumulation in cells was also observed on a fluorescence microscope (BX51 with UC30 camera; Olympus Corporation, Tokyo, Japan).

### 4.7. Evaluation of the Endogenous Antioxidant Defense System through GSH Levels

Based on the methodology adapted from Daré [57], MCF-7 and MDA-MB-231 cells plated in 6-well plates were treated with **BCP-1**. H_2_O_2_ (200 µM) was used as a PC. Cell lysates were obtained by scraping the monolayers using ice-cold lysis buffer (10 mM Tris-HCl, pH 7.4, and 1% Triton X-100), followed by sonication for 60 s (30% amplitude, 5 s on and 5 s off) and centrifugation at 10,000× *g* for 10 min at 4 °C. The amount of protein was estimated with Bradford’s reagent and cell lysates were stored at −80 °C until assayed. In 96-well black plates, 180 μL of sodium phosphate buffer (100 mM, 5 mM EDTA, pH 8.0), 10 μL of cell lysate, and 10 μL of OPA(1 mg/mL in methanol) were added and the fluorescence was determined after 15 min of incubation at room temperature in a microplate reader (VICTOR™ X3; PerkinElmer, MA, USA) at excitation/emission wavelengths of 350/420 nm, respectively. The results were expressed according to the calibration curve of the GSH standard (1953–1000 μg/mL).

### 4.8. Mitochondrial Membrane Potential Assessment (ΔΨm)

MCF-7 and MDA-MB-231 cells plated in 24-well plates were treated with **BCP-1**. CCCP (100 μM), a mitochondrial membrane uncoupler, was used as a PC. Cells were resuspended in PBS and then labeled with the red-orange cationic marker TMRE (25 nm) for 30 min at room temperature. Fluorescence was measured in a microplate reader (VICTOR™ X3; PerkinElmer, MA, USA) at excitation/emission wavelengths of 485/535 nm, respectively [54].

### 4.9. Quantification of Intracellular Adenosine Triphosphate (ATP) Levels

MCF-7 and MDA-MB-231 cells plated in 24-well plates were treated with **BCP-1**. KCN (1000 μM), a molecule responsible for inhibiting cell respiration, was used as a PC. Cells were trypsinized, resuspended in PBS, and labeled with 50 μL of CellTiter-Glo^®^ reagent for 10 min at room temperature. This reagent was used to determine the number of viable cells by quantifying the ATP present. Luminescence was quantified in a microplate reader (VICTOR™ X3; PerkinElmer, MA, USA).

### 4.10. Cell Membrane Assessment

#### 4.10.1. Evaluation of Lipid Peroxidation

Following the methodology of Kaplum [58], MCF-7 and MDA-MB-231 cells plated in 24-well plates were treated with **BCP-1**. H_2_O_2_ (200 µM) was used as a PC. Cells were then trypsinized, resuspended in PBS, and labeled with DPPP (50 µM) for 15 min at room temperature. Fluorescence was quantified in a microplate reader (VICTOR™ X3; PerkinElmer, MA, USA) with excitation/emission wavelengths of 351/380 nm, respectively.

#### 4.10.2. Assessment of Cell Membrane Integrity

MCF-7 and MDA-MB-231 cells plated in 24-well plates were treated with **BCP-1**. Digitonin (50 μM), a reagent that solubilizes membrane proteins, was used as a PC. Cells were then trypsinized, resuspended in PBS, and labeled with PI (4 μg/mL) for 5 min at room temperature. The fluorescence intensity was quantified in a microplate reader (VICTOR™ X3; PerkinElmer, MA, USA) with excitation/emission wavelengths of 480/580 nm, respectively.

### 4.11. Cell cycle Analysis

Following the methodology of Lazarin-Bidóia [59], MCF-7 and MDA-MB-231 cells cultured in 6-well plates were treated with **BCP-1**. Cells were then washed with PBS, trypsinized, and fixed in 70% methanol at 4 °C for 1 h. Following this, cells were washed and incubated in PBS supplemented with PI (10 µg/mL) and RNAse A (20 µg/mL) at 37 °C for 45 min. Data acquisition and analysis were performed on a FACSCalibur flow cytometer (Becton-Dickinson, Rutherford, NJ, USA) equipped with CellQuest software (Joseph Trotter, Scripps Research Institute, La Jolla, CA, USA). A total of 10,000 events were acquired. The percentage of cells at each stage of the cell cycle was determined using the FlowJo_v10 software.

### 4.12. Cell Chromatin Analysis

MCF-7 and MDA-MB-231 cells were plated on round coverslips placed in 24-well plates and treated with **BCP-1**. CPT (10 μM) was used as a PC. Cells were washed in PBS, stained with the Hoechst 33342 marker (2 μg/mL) and incubated for 20 min at 37 °C in the dark. Following this, cells were washed twice with PBS and analyzed under a fluorescence microscope (BX51 with UC30 camera; Olympus Corporation, Tokyo, Japan). The identification criteria were as follows: cells that had homogeneously stained nuclei (light blue) were considered live, while cells with intense staining (bright blue) indicated apoptosis due to chromatin condensation. The fluorescence intensity was determined by the ImageJ software [56].

### 4.13. Evaluation of DNA Fragmentation by Agarose Gel Electrophoresis

MCF-7 and MDA-MB-231 cells plated in 6-well plates were treated with **BCP-1**. CPT (30 μM) was used as a PC. Cells were lysed in a solution containing Tris-HCl (10 mM; pH 8), EDTA (1 mM), NaCl (100 mM), SDS (0.5%), and proteinase k (20 mg/mL) at 65 °C for 15 min. RNAse (1 mg/mL) was then added for 15 min at 37 °C. DNA was purified using phenol:chloroform:isoamylalcohol (25:24:1; *v*/*v*). DNA electrophoresis was performed on a 0.75% agarose gel in Tris/boric acid buffer at 90 V for 1 h, and the DNA was stained with SYBR Safe (Invitrogen, MA, USA). The molecular weight marker used was the 100 bp DNA Ladder. The images were obtained using the ChemiDoc^®^ XRS + Imaging System (Bio-Rad Laboratories Inc., Hercules, CA, USA) [56].

### 4.14. Detection of Phosphatidylserine Exposure

MCF-7 and MDA-MB-231 cells plated in 6-well plates were treated with **BCP-1**. Cells were then dissociated, washed with PBS, and resuspended in 100 µL of binding buffer (140 mM NaCl, 5 mM CaCl_2_, 10 mM HEPES-Na, pH 7.4), followed by the addition of 5 µL Annexin V-FITC for 15 min at room temperature. Afterwards, 400 µL of binding buffer and 50 µL of PI were added. Data acquisition and analysis were performed using a FACSCalibur flow cytometer (Becton-Dickinson, Rutherford, NJ, USA) equipped with CellQuest software (Joseph Trotter, Scripps Research Institute, La Jolla, CA, USA). A total of 10,000 events were acquired. The identification criteria were as follows: annexin V-positive cells were considered to be cells in early apoptosis, annexin V/PI-positive cells were considered to be in late apoptosis, PI-positive cells were considered to be necrotic cells, and cells that were not positive for any of the markers were considered viable cells [60].

### 4.15. Evaluation of the Type of Cell Death by Double Labeling with AO/PI

MDA-MB-231 cell monolayers plated on round coverslips in 24-well plates were treated with **BCP-1**. CPT (20 µM) was used as a PC. Cells were then labeled with AO (10 μg/mL) and PI (4 μg/mL) for 15 min in the dark. Fluorescence was analyzed on a fluorescence microscope (BX51 with UC30 camera; Olympus Corporation, Tokyo, Japan) within 30 min. The identification criteria were as follows: viable cells presented green nuclei with intact structures; cells in early apoptosis had a bright green nucleus indicating chromatin condensation; and cells in late apoptosis showed dense orange areas indicative of condensed chromatin and reddish-orange nuclei indicating necrosis. The number of viable, early apoptotic, late apoptotic, and necrotic cells were determined by counting 200 cells in triplicate [58].

### 4.16. Western Blotting

MCF-7 and MDA-MB-231 cells were cultured in 6-well plates and treated with **BCP-1**. After treatment, cells were detached using a cell scraper and centrifuged at 1000× *g* at 4 °C for 10 min. The obtained pellet was resuspended in ice-cold lysis buffer [150 mM NaCl, 5 mM EDTA, 50 mM Tris-HCl (pH 8.0), 1% Triton X-100, 5% SDS, and 1% protease inhibitor cocktail]. Cell lysates were sonicated for 2 min (Branson Ultrasonics^®^; 60% amplitude, 20 s on and 20 s off) and centrifuged at 10,000× *g* for 20 min at 4 °C. The amount of protein was estimated with Bradford’s reagent. Samples of equivalent amounts of protein were heated at 100 °C for 5 min in loading buffer [5% mercaptoethanol, 5% bromophenol blue, 75 mM Tris-HCl (pH 6.8), 2% SDS, and 10% glycerol]. Proteins were subjected to 12% SDS-polyacrylamide gel electrophoresis, then transferred to a nitrocellulose membrane in transfer buffer (25 mM Tris base, 192 mM glycine, 20% methanol, and 0.01% SDS). The primary antibodies used for MDA-MB-231 cells were mouse anti-Bax, mouse anti-Bcl-2, mouse anti-cytochrome C, mouse anti-caspase-3, mouse anti-caspase-9 (1:500), mouse anti-MMP-9 (1:400), and mouse anti-β-actin (1:10,000). The primary antibodies used for MCF-7 cells were mouse anti-LC3I/LC3II, mouse anti-p62/SQSTM1 (1:500), and mouse anti-β-actin (1:10,000); all of which were diluted in TBS-T buffer with 3% albumin. Membranes were washed and incubated with mouse secondary antibody M-IGGk BP-HRP (1:5000) for 1 h at room temperature. The antigen-antibody reaction was detected by chemiluminescence with the ECL detection reagent (Santa Cruz Biotechnology, CA, USA) and analyzed on a ChemiDoc^®^ XRS + Imaging System (Bio-Rad Laboratories Inc., Hercules, CA, USA) [57].

### 4.17. Assessment of Autophagic Vacuoles

MCF-7 cells cultured in 24-well plates were treated with **BCP-1**. Subsequently, wortmannin (1 µM) was added, a potent and selective inhibitor of phosphatidylinositol 3-kinase (PI3K), a family of enzymes necessary for autophagy [20]. After the treatments, cells were washed with PBS and incubated with MDC marker (0.05 mM) in PBS for 10 min in the dark. Fluorescence intensity was measured at the excitation/emission wavelengths of 335/512 nm, respectively, in a microplate reader (VICTOR™ X3; PerkinElmer, MA, USA).

### 4.18. Evaluation of Acidic Organelles

Based on the methodology of Cui [11], MCF-7 cells were plated in 24-well plates and treated with **BCP-1**. Cells were washed with PBS and the LysoTracker^®^ red marker (50 nm) was added, followed by incubation for 30 min in the dark. Fluorescence was measured at excitation/emission wavelengths of 510/560 nm, respectively, in a microplate reader (VICTOR™ X3; PerkinElmer, MA, USA).

### 4.19. Cell Migration Assay

MCF-7 and MDA-MB-231 cell monolayers were cultured in 24-well plates for 24 h. Then, the contents of the wells were removed and replaced by DMEM supplemented with 0.5% FBS and incubated for 6 h at 37 °C and 5% CO_2_. Subsequently, a wound was made in the cell monolayer by scraping with a 200 μL tip. Following this, the cells were treated, or not, with **BCP-1**. At 0, 24, and 48 h, the scars were visualized under an inverted phase contrast microscope (4× *g* magnification; Olympus CKX41; Olympus Corporation, Tokyo, Japan) coupled with a US-30 camera. The percentage of cell growth in the scarred region was evaluated using ImageJ software to determine cell migration capacity [58].

### 4.20. Clonogenic Assay

MCF-7 and MDA-MB-231 cells cultivated in 6-well plates were treated with **BCP-1**. Subsequently, the treatment medium was removed, and cells were incubated with pure culture medium (DMEM) for ten days. The medium was renewed every 72 h. Following this, the colonies formed were washed with PBS, fixed in ice-cold methanol (100%) for 10 min, washed again with PBS, and stained with 5% Giemsa for 45 min. The number of cells was counted, and a colony was considered when there were more than 50 cells. Colonies were observed using an inverted microscope (4× *g* magnification; Olympus CKX41; Olympus Corporation, Tokyo, Japan) coupled with a US-30 camera [61].

### 4.21. Statistical Analysis

Statistical analyses were performed using GraphPad Prism software v. 8.00 (San Diego, CA, USA). The data shown in tables and graphs express the mean ± standard deviation (SD) of at least three independent experiments. Data were analyzed using ANOVA tests (one-way or two-way), followed by Tukey’s post-test. Differences were statistically significant when *p* < 0.05.

## 5. Conclusions

This in vitro investigation of the effects of **BCP-1** on breast cancer cell lines demonstrated that there is a selective action of this compound on the proliferation of MCF-7 and MDA-MB-231 tumor cells in relation to normal MCF-10A cells. **BCP-1** induced oxidative stress and mitochondrial dysfunction, in addition to decreasing intracellular levels of ATP, consequently leading to cell death. MDA-MB-231 cells treated with **BCP-1** showed induction of the apoptotic death pathway, as demonstrated by the annexin V/PI and AO/PI labeling, in addition to the regulation of proteins related to apoptosis. On the other hand, MCF-7 cells presented induction of the autophagic death pathway, owing to the accumulation of autophagic vacuoles and acidic organelles, and the regulation of the expression of specific autophagy proteins. These findings highlight **BCP-1** as a promising candidate in the development of a therapeutic agent for breast cancer. Thus, our data support future studies aimed at analyzing other compounds derived from **BCP-1** that possess β-carboline and α-aminophosphonate moieties, in addition to carrying out in vivo, preclinical, and clinical trials of **BCP-1** to further validate the antitumor effects of this compound in breast cancer.

## Figures and Tables

**Table 1:** Cell viability of MCF-7, MDA-MB-231, and MCF-10A cell lines after treatment with **BCP-1** for 24 and 48 h.

Cell Line	IC_50_ 24 h (µM)	IC_50_ 48 h (µM)	SI 24 h	SI 48 h
MCF-7	50.2 ± 3.0	32.3 ± 1.0	2.96	3.62
MDA-MB-231	66.7 ± 3.2	38.6 ± 1.1	2.23	3.03
MCF-10A	149.2 ± 4.2	117.3 ± 3.4	___	___

IC_50_ = Inhibitory concentration for 50% treated cells compared to the untreated cells. SI = Selectivity Index.

## Data Availability

The data presented in this study are available in this manuscript.
